# Factors associated with hospitalizations for Covid-19 in patients with rheumatoid arthritis: data from the Reumacov Brazil registry

**DOI:** 10.1186/s42358-022-00244-5

**Published:** 2022-05-03

**Authors:** Ana Paula Monteiro Gomides, Cleandro Pires de Albuquerque, Licia Maria Henrique da Mota, Guilherme Devidé, Laiza Hombre Dias, Angela Luzia Branco Pinto Duarte, Raquel Altoé Giovelli, Thais Evelyn Karnopp, Hugo Deleon de Lima, Adriana Marinho, Marianne Schrader de Oliveira, Felipe Omura, Aline Ranzolin, Gustavo Resende, Francinne Machado Ribeiro, Sandra Lúcia Euzébio Ribeiro, Nathália de Carvalho Sacilotto, Wander Gonzaga dos Santos, Samuel Katsuyuki Shinjo, Samia Araujo de Sousa Studart, Flávia Patricia Sena Teixeira, Michel Alexandre Yazbek, Gilda Aparecida Ferreira, Odirlei A. Monticielo, Eduardo Paiva, Gecilmara Cristina Salviato Pileggi, Edgard Torres dos Reis-Neto, Marcelo de Medeiros Pinheiro, Claudia D. L. Marques, On behalf of ReumaCoV Brasil Registry

**Affiliations:** Faculdade de Ciências da Educação e Saúde, Centro Universitário de Brasília, Brasília, Brazil

**Keywords:** COVID-19, Hospitalization, Immunosuppression, Outcomes, Rheumatoid arthritis

## Abstract

**Background:**

Patients using immunosuppressive drugs may have unfavorable results after infections. However, there is a lack of information regarding COVID-19 in these patients, especially in patients with rheumatoid arthritis (RA). Therefore, the aim of this study was to evaluate the risk factors associated with COVID-19 hospitalizations in patients with RA.

**Methods:**

This multicenter, prospective cohort study is within the ReumaCoV Brazil registry and included 489 patients with RA. In this context, 269 patients who tested positive for COVID-19 were compared to 220 patients who tested negative for COVID-19 (control group). All patient data were collected from the Research Electronic Data Capture database.

**Results:**

The participants were predominantly female (90.6%) with a mean age of 53 ± 12 years. Of the patients with COVID-19, 54 (20.1%) required hospitalization. After multiple adjustments, the final regression model showed that heart disease (OR = 4.61, 95% CI 1.06–20.02. *P* < 0.001) and current use of glucocorticoids (OR = 20.66, 95% CI 3.09–138. *P* < 0.002) were the risk factors associated with hospitalization. In addition, anosmia was associated with a lower chance of hospitalization (OR = 0.26; 95% CI 0.10–0.67, *P* < 0.005).

**Conclusion:**

Our results demonstrated that heart disease and the use of glucocorticoids were associated with a higher number of hospital admissions for COVID-19 in patients with RA.

*Trial registration*: Brazilian Registry of Clinical Trials - RBR-33YTQC.

**Supplementary Information:**

The online version contains supplementary material available at 10.1186/s42358-022-00244-5.

## Background

The COVID-19 pandemic has raised additional concerns for rheumatologists, especially related to health care for patients with immune-mediated rheumatic diseases (IMRDs) [1, 2]. Notably, immunosuppression resulting from disease or treatment itself is considered a relevant risk factor for higher infection susceptibility and more severe outcomes [3].

Although some evidence on the course of SARS-CoV-2 infections in patients with IMRDs has already been demonstrated, such as a similar COVID-19 prevalence to that of the general population [4], there are some knowledge gaps and uncertainties regarding the peculiarities and possible unfavorable outcomes, including hospitalization rate, frequency of admission to intensive care centers and severity [5–11]. In addition, immunosuppressive treatments for underlying diseases, glucocorticoids, and cyclophosphamide may contribute to unfavorable outcomes [12, 13]. On the other hand, a hypothesis has been presented that some immunomodulatory drugs, such as conventional or biological disease-modifying anti-rheumatic drugs (DMARDs), can help to mitigate the inflammation and cytokine storm induced by SARS-CoV-2 [14, 15].

Considering the rheumatoid arthritis (RA) treatment scenario, which includes disease activity, low-dose prednisone, methotrexate, TNF blockers, IL-6 antagonists, JAK inhibitors, and anti-CD20 therapy, as a prototype for these findings, this study had the main aim of evaluating the risk factors associated with COVID-19 hospitalizations in patients with RA.

## Methods

This was a cross-sectional analysis performed only in patients with RA who were enrolled in the ReumaCoV-Brazil Registry, a multicenter, prospective cohort study, used to evaluate and monitor patients with IMRDs during the COVID-19 pandemic. The complete methodology and initial data were published previously [12, 16]. Eligible patients were selected through a convenience sample after the researchers contacted patients with RA and COVID-19 via telephone calls and noted similar patients in outpatient care or hospitals. They then compared the patients with RA who had COVID-19 with patients with RA who did not have COVID-19.

The following inclusion criteria were considered: (a) an age older than 18 years; (b) a diagnosis of COVID-19, according to the Ministry of Health; and (c) a previous diagnosis of RA, according to the American College of Rheumatology or the European League against Rheumatism criteria. The exclusion criteria were patients with HIV or other immunodeficiency diseases, previous organ or bone marrow transplants, neoplasms in the last five years, current chemotherapy treatment and diseases of the thymus.

The Research Electronic Data Capture (REDCap) (https://www.project-redcap.org/) electronic database was used to collect and record the patients’ sociodemographic aspects and information on RA (time of disease, disease activity, laboratory data, use of DMARDs and other concomitant medications, comorbidities and details about the COVID-19 infection (clinical manifestations, treatment and outcomes)). Information was obtained by telephone or a face-to-face consultation, according to the local recommendations due to the pandemic, and through medical records in the event of hospitalization.

### Statistical analysis

Initially, the data were analyzed descriptively. For categorical variables, absolute and relative frequencies were presented, and for numerical variables, summary measures (mean, median, minimum, maximum and standard deviation) were presented. For associations among categorical variables, the chi-square test was used, or Fisher’s exact test was used when cells had expected values of less than five counts. In cases of discrepancies, the standardized adjusted residue was used to identify local differences–cells with absolute values above 1.96 indicated significant deviations from expected values assignable only to random variations.

The comparisons of the means between two groups and more than two groups were performed using Student’s t-test for independent samples and analysis of variance (ANOVA), respectively. Normality in data distribution was verified by the Kolmogorov–Smirnov test. In the event of assumption violations to Student’s t-test and ANOVA, the Mann–Whitney and Kruskal–Wallis nonparametric tests were used, respectively. Once the Kruskal–Wallis test was significant, the location of paired differences was determined by Dunn Bonferroni tests, maintaining an overall significance level of 5%.

To assess the simultaneous effects of the demographic and clinical characteristics (e.g., predictor variables) on hospitalizations (e.g., dependent variable), univariate and multivariate logistic regressions were adjusted. For the initial multivariate models, predictor variables whose associations with the dependent variable were significant at 10% in the univariate logistic regression were selected. Then, a backward procedure was conducted by excluding the variables one by one in order of significance that were not found to be more significant at 5% in the multivariate regression than those in the final model. The final model fit was then evaluated by a Hosmer and Lemeshow test. Due to the large number of predictor variables and the small number of events, the variables whose associations with the dependent variable were significant amounted to 10% in the univariate logistic regression. Then, the variables that were not significant to 5% were excluded one by one in order of significance (backward method). In addition, the adjustment adequacy of the final model was evaluated by the Hosmer and Lemeshow test.

For all statistical tests, a significance level of 5% was used. Statistical analyses were performed using SPSS 20.0 statistical software.

This study was approved by the Brazilian Committee of Ethics in Human Research on April 5, 2020, (CAAE 30,186,820.2.1001.8807) and registered on the Brazilian Registry of Clinical Trials (RBR-33YTQC) on June 1, 2020. All patients signed informed consent forms, and the results of the study are presented in an aggregated form, guaranteeing confidentiality and ensuring that there are no risks to patients’ well-being and care.

## Results

From May 24, 2020, to January 31, 2021, 489 patients with RA were included: 269 tested positive for COVID-19, and 220 tested negative for COVID-19 (control group). There was a female predominance (n = 442; 90.6%) with a mean age of 53 ± 12 years. Considering only the patients with COVID-19, 54 (20.1%) patients required hospitalization.

Comparing hospitalized patients with COVID-19 with outpatients, patients with COVID-19 were older, and had one, two or more comorbidities present. Diabetes mellitus and hypertension were significantly more prevalent in those who required hospital care (Table 1). In addition, patients with shortness of breath, cough and vomiting were significantly more likely to be hospitalized. On the other hand, patients with anosmia and dysgeusia had a lower hospitalization rate (Table 2).

**Table 1:** General characteristics and comorbidities of patients with rheumatoid arthritis and COVID-19

Hospitalization	No	Yes	*P*
	N = 215	N = 54	
Female, n (%)	196/214 (91.6)	50/54 (92.6)	0.810
Age (years), mean (SD)	51.9 (12.2)	58.6 (10.6)	< 0.001^b^
Profession			0.904
Customer Service n (%)	32/211 (15.2)	6/52 (11.5)	
Health n (%)	23/211 (10.9)	5/52 (9.6)	
Education n (%)	17/211 (8.1)	6/52 (11.5)	
Housewife n (%)	59/211 (28.0)	15/52 (28.8)	
Others n (%)	80/211 (37.9)	20/52 (38.5)	
Active work situation	120/212 (56.6)	25/54 (46.3)	0.174
Comorbidities	139/214 (65.0)	46/54 (85.2)	0.004
Heart disease	10/214 (4.7)	7/54 (13.0)	0.053^a^
Diabetes mellitus	20/214 (9.3)	11/54 (20.4)	0.024
Lung disease	15/214 (7.0)	2/54 (3.7)	0.538^a^
Kidney disease	4/214 (1.9)	2/54 (3.7)	0.348^a^
Systemic arterial hypertension	82/214 (38.3)	32/54 (59.3)	0.005
Obesity	30/214 (14.0)	12/54 (22.2)	0.138
Others	73/214 (34.1)	25/54 (46.3)	0.097
Number of comorbidities			0.003
None n (%)	75/214 (35)	8/54 (14.8)	
1 comorbidity n (%)	74/214 (34.6)	18/54 (33.3)	
2 or more comorbidities n (%)	65/214 (30.4)	28/54 (51.9)	
Smoking	16/213 (7.5)	2/54 (3.7)	0.542^a^

*P*—Chi-Square test, Fisher's exact (^a^) or Student's t (^b^)

**Table 2:** Symptoms related to COVID-19 in patients with rheumatoid arthritis

Parameters	Hospitalization		*P*
	No	Yes	
	N = 215	N = 54	
Asymptomatic	14/215 (6.5)	1/54 (1.9)	0.318^a^
Skin manifestations	7/215 (3.3)	1/54 (1.9)	1.000^a^
Arthralgia	71/215 (3.03)	24/54 (44.4)	0.116
Asthenia	107/215 (49.8)	28/54 (51.9)	0.784
Headache	136/215 (63.3)	32/54 (59.3)	0.588
Rhinorrhea	66/215 (30.7)	14/54 (25.9)	0.493
Diarrhoea	76/215 (35.3)	20/54 (37.0)	0.817
Dyspnoea	75/215 (34.9)	39/54 (72.2)	< 0.001
Fever	112/215 (52.1)	31/54 (57.4)	0.484
Myalgia	98/215 (45.6)	34/54 (63.0)	0.022
Nausea	50/215 (23.3)	16/54 (29.6)	0.33
Anosmia	126/215 (58.6)	18/54 (33.3)	0.001
Ageusia	123/215 (57.2)	20/54 (37.0)	0.008
Dizziness	44/215 (20.5)	8/54 (14.8)	0.347
Cough	106/215 (49.3)	38/54 (70.4)	0.006
Vomits	31/215 (14.4)	15/54 (27.8)	0.02
Other symptoms	53/215 (24.7)	12/54 (22.2)	0.709

*P*—Chi-Square test, Fisher's exact (^a^) or Student's *t*

Considering specific findings related to RA, including autoantibody status and cumulative damage (erosions), extra-articular manifestations, withdrawal therapy, and the current use of azathioprine and corticosteroids were associated with hospitalization. On the other hand, patients on TNF inhibitors had a significantly lower frequency of hospitalization (Tables 3 and 4).

**Table 3:** Characteristics of rheumatoid arthritis in the studied population

	Without hospitalization	Hospitalization	*P*
Rheumatoid factor	95/115 (82.6)	20/115 (17.4)	0.059
Anti-CCP	34/45 (75.6)	11/45 (24.4)	0.255
Erosive disease	60/82 (73.2)	22/82 (26.8)	0.089
Extra-articular manifestations	13/22 (59.1)	9/22 (40.9)	0.030^a^

*P*—descriptive level of the Chi-Square test, Fisher's exact (^a^) or Student's *t*

*Anti-CCP* anti-cyclic cirullinated peptide antibodies, *SD* Standard deviation

**Table 4:** Disease-modifying antirheumatic or immunossupressive drugs used by patients with rheumatoid arthritis at the time of the study

Hospitalization	No	Yes	*P*
	N = 215	N = 54	
*Disease-modifying antirheumatic or immussupressive drugs*			
Without treatment			1.000^a^
No n (%)	210/215 (97.7)	53/54 (98.1)	
Yes n (%)	5/215 (2.3)	1/54 (1.9)	
Abatacept			0.202^a^
No n (%)	210/215 (97.7)	51/54 (94.4)	
Yes n (%)	5/215 (2.3)	3/54 (5.6)	
IL-17 inhibitors?			–
No n (%)	215/215 (100.0)	54/54 (100.0)	
IL12/23 inhibitors?			1.000^a^
No n (%)	214/215 (99.5)	54/54 (100.0)	
Yes n (%)	1/215 (0.5)	0/54 (0.0)	
TNF inhibitors			0.014
No n (%)	147/215 (68.4)	46/54 (85.2)	
Yes n (%)	68/215 (31.6)	8/54 (14.8)	
Azathioprine			0.040^a^
No n (%)	215/215 (100.0)	52/54 (96.3)	
Yes n (%)	0/215 (0.0)	2/54 (3.7)	
Belimumab?			–
No n (%)	215/215 (100.0)	54/54 (100.0)	
Cyclophosphamide oral			–
No n (%)	215/215 (100.0)	54/54 (100.0)	
Ciclosporin			–
No n (%)	215/215 (100.0)	54/54 (100.0)	
Corticosteroids (oral)			0.004^a^
No use	149/215 (69.3)	26/54 (48.1)	
< 10 mg/day n (%)	58/215 (27.0)	20/54 (37.0)	
≥ 11 to 20 mg/day n (%)	6/215 (2.8)	7/54 (13.0)	
≥ 21 mg/day n (%)	2/215 (0.9)	1/54 (1.9)	
Hydroxychloroquine			0.910
No n (%)	194/215 (90.2)	49/54 (90.7)	
Yes n (%)	21/215 (9.8)	5/54 (9.3)	
JAKi			0.789^a^
No n (%)	197/215 (91.6)	49/54 (90.7)	
Yes n (%)	18/215 (8.4)	5/54 (9.3)	
Leflunomide			0.791
No n (%)	163/215 (75.8)	40/54 (74.1)	
Yes n (%)	52/215 (24.2)	14/54 (25.9)	
Methotrexate			0.783
No use n (%)	117/211 (55.5)	32/54 (59.3)	
≤ 20 mg/week n (%)	77/211 (36.5)	17/54 (31.5)	
≥ 21 mg/week n (%)	17/211 (8.1)	5/54 (9.3)	
Mycophenolatemofetil			–
No n (%)	215/215 (100.0)	54/54 (100.0)	
Cyclophosphamide pulsotherapy			–
No n (%)	215/215 (100.0)	54/54 (100.0)	
Methylprednisolone (pulsotherapy)			–
No n (%)	215/215 (100.0)	54/54 (100.0)	
Rituximab			0.490^a^
No n (%)	205/215 (95.3)	50/54 (92.6)	
Yes n (%)	10/215 (4.7)	4/54 (7.4)	
Sulfasalazine			0.346^a^
No n (%)	211/215 (98.1)	52/54 (96.3)	
Yes n (%)	4/215 (1.9)	2/54 (3.7)	
Tocilizumab			0.687
No n (%)	191/215 (88.8)	49/54 (90.7)	
Yes n (%)	24/215 (11.2)	5/54 (9.3)	
Others			0.283
No n (%)	183/215 (85.1)	49/54 (90.7)	
Yes n (%)	32/215 (14.9)	5/54 (9.3)	
scDMARD			0.878
No n (%)	62/215 (28.8)	15/54 (27.8)	
Yes n (%)	153/215 (71.2)	39/54 (72.2)	
bDMARD			0.083
No n (%)	107/215 (49.8)	34/54 (63.0)	
Yes n (%)	108/215 (50.2)	20/54 (37.0)	
Withdrawal			0.010
No n (%)	168/215 (78.1)	33/54 (61.1)	
Yes n (%)	47/215 (21.9)	21/54 (38.9)	

*P*—Chi-Square test, Fisher's exact (^a^) or Student's t

After multiple adjustments, the final regression model showed that the risk factors significantly associated with hospitalization were shortness of breath (OR 6.12; 95% CI 2.34–16.06, *P* < 0.001), vomiting (OR 4.06; 95% CI 1.4–11.79, *P* < 0.01), heart disease (OR 4.61; 95% CI 1.06–20.02, *P* < 0.001), and the current use of glucocorticoids (OR 20.66; 95% CI 3.09–138, *P* < 0.002). Moreover, anosmia was associated with a lower chance of hospitalization (OR 0.26; 95% CI 0.10–0.67, *P* < 0.005) (Table 5). The results of the univariate logistic regression model are presented in the Additional file 1.

**Table 5:** Multivariate logistic regression models for the outcome variable hospitalization in patients with rheumatoid arthritis and covid-19”

Predictor variables	Initial model		Final model	
	Adjusted OR (95% CI)	*P*	Adjusted OR (IC95%CI)	*P*
Age (years)	1.03 (0.98–1.08)	0.275	–	–
Comorbidities				
No comorbidity	1.27 (0.19–8.38)	0.805	–	–
Heart disease	8.65 (1.12–66.54)	0.038	4.61 (1.06–20.02)	0.041
Diabetes mellitus	1.44 (0.26–7.88)	0.675	–	–
Hypertension	2.18 (0.61–7.75)	0.230	–	–
Other	2.3 (0.67–7.83)	0.185	–	–
Symptoms				
Dyspnoae	7.47 (2.15–25.95)	0.002	6.12 (2.34–16.06)	< 0.001
Myalgia	0.57 (0.17–1.89)	0.357	–	–
Anosmia	0.24 (0.06–0.92)	0.037	0.26 (0.10–0.67)	0.005
Ageusia	1.13 (0.23–5.50)	0.884	–	–
Cough	0.9 (0.27–2.99)	0.859	–	–
Vomits	4.65 (1.4–15.47)	0.012	4.06 (1.4–11.79)	0.01
*Type of confirmatory examination*				
RT-PCR (ref. = Not diagnosed)		< 0.001		< 0.001
No	1.96 (0.29–13.35)	0.490	1.46 (0.30–7.14)	0.644
Yes	26.27 (4.74–145.53)	< 0.001	11.54 (3.28–40.59)	< 0.001
Drugs				
Anti-TNF	0.65 (0.13–3.22)	0.595	–	–
Oral corticosteroid (ref. = no use)		0.005		0.016
< 10 mg/day	1.44 (0.45–4.6)	0.538	1.89 (0.73–4.94)	0.192
≥ 11 to 20 mg/day	71.39 (6.79–750.92)	< 0.001	20.66 (3.09–138.0)	0.002
≥ 21 mg/day	6.21 (–)	0.840	2.25 (-)	0.832
bDMARD	1.38 (0.38–5.01)	0.626		
Treatment suspension	1.61 (0.53–4.91)	0.401	–	–
*Drugs used to treat COVID-19*				
No drug	0.2 (0.01–3.64)	0.277	–	–
Azytromycin	1,88 (0,55—6,49)	0.317	–	–
Oral corticosteroid (ref. = no use)		0.178	–	–
< 10 mg/day	7.22 (1.19–43.81)	0.032	–	–
≥ 11 to 20 mg/day	0.96 (0.19–4.93)	0.964	–	–
≥ 21 mg/day	0.72 (0.09–5.76)	0.753	–	–
Heparin	20.58 (3.39–124.81)	0.001	15.89 (4.27–59.11)	< 0.001
N	261		261	

(–) without precision

*OR* odds ratio, *95% CI* 95% confidence interval, *bDMARD* biologic DMARD

## Discussion

Our data demonstrated that approximately 20% of patients with RA required hospitalization because of COVID-19, a rate lower than that in another large registry study (38%) [13] but similar to the second analysis from the same database (21%) [17]. The findings suggest that other details beyond disease alone could be involved, including genetic background and epidemiological differences (e.g., spreading time or viral community transmission) among the countries.

The main advantage of our study was the inclusion of a large sample of patients with RA. In addition, all data were preparametrized, prestandardized and captured by a trained rheumatologist and with laboratory test confirmation for those who tested positive for COVID-19.

Considering the traditional risk factors related to COVID-19 severity, our results did not find a significant association with age after multiple adjustments for cofounders, which is contrary to other studies from the general population and IMRD cohorts [13, 18–21].

However, heart disease, as a comorbidity, was significantly associated with hospitalization, which is in accordance with the current literature [22–33].

These results suggest that the comorbidity burden seems to be similar to several reports from the general population, regardless of underlying rheumatic disease [13, 31–41].

Although some clinical findings were associated with hospitalization in our cohort, such as shortness of breath and vomiting, these findings may be redundant because they are considered red flags or parameters for hospitalization [37–48]. Therefore, this particular result should be interpreted with caution, especially in a cross-sectional analysis.

Interestingly, anosmia, an important specific clinical marker for COVID-19 [48], had a protective behavior against hospitalization, as previously reported by other authors [44–50]. Thus, our data suggest that anosmia could be seen as a potential clinical marker of mild COVID-19 severity in patients with RA.

It is worth emphasizing that the clinical and laboratory characteristics of RA and DMARDs (e.g., conventional, biological and targeted synthetic medications) were not associated with hospitalization in the final analysis, except for the current use of glucocorticoids (dosage equal to or greater than 10 mg/day). This is a recurrent finding among the studies [12, 13].

Patients with RA on TNF inhibitors had a lower rate of hospitalizations in the initial analysis but this finding was not confirmed after multivariate analysis which is contrary to own findings when considering the entire sampling of patients with IMRDs [12] and the Global Alliance Rheumatology (GRA) database [13, 51] and another study published in the literature [52].

In addition, the second analysis of the GRA database that involved 2869 patients with RA found that rituximab (OR = 4.15; 95% CI 3.16–5.44) and JAK inhibitors (OR = 2.06; 95% CI 1.60–2.65) were significantly associated with COVID-19 severity [17]. Our data did not confirm the last reports.

Our study has some limitations, such as the convenience sample and small number of patients, cross-sectional analysis of an ongoing prospective cohort, the low number of rituximab users and specific DMARD targets and the lack of information on disease activity. On the other hand, our study included a national sample with a more homogeneous rate of community viral transmission, social distancing measures and immunization.

## Conclusions

Our data showed traditional risk factors, including heart disease as a comorbidity, and the current use of glucocorticoids are more involved with hospitalizations for COVID-19 in patients with RA than the underlying IMRDs alone.

## Supplementary Information

**Additional file 1.** Results of univariate logistic regression models - hospitalization in patients with rheumatoid arthritis.

## Data Availability

The datasets used and/or analysed during the current study are available from the corresponding author on reasonable request.

## Abbreviations

RA: Rheumatoid arthritis
IMRDS: Immune-mediated rheumatic diseases
DMARDS: Disease-modifying anti-rheumatic drugs
