# Supplementary material for: Factors associated with hospitalizations for Covid-19 in patients with rheumatoid arthritis: data from the Reumacov Brazil registry
Source: Adv Rheumatol. 2022 May 3;62(1):13. doi: 10.1186/s42358-022-00244-5 (PMC9062867; doi:10.1186/s42358-022-00244-5)
Supplement: Supplementary file 1 — Additional file 1. Results of univariate logistic regression models - hospitalization in patients with rheumatoid arthritis. [file 42358_2022_244_MOESM1_ESM.docx]

Table supplementary material-

**Results of the univariate logistic regression models - hospitalization in patients with rheumatoid arthitis**

|  | **OR (IC95%)** | **p** |
| --- | --- | --- |
| **Male gender** | 0,87 (0,28 - 2,69) | 0,810 |
| **Age (years)** | 1,05 (1,02 - 1,08) | <0,001 |
| **Profession** |  | 0,906 |
| Customer service | 0,75 (0,28 - 2,04) | 0,573 |
| Health | 0,87 (0,29 - 2,57) | 0,801 |
| Education | 1,41 (0,49 - 4,04) | 0,520 |
| Housewife | 1,02 (0,48 - 2,15) | 0,965 |
| **Inactive work situation** | 1,51 (0,83 - 2,76) | 0,176 |
| **Comorbidities** |  |  |
| No comorbidity | 0,32 (0,15 - 0,72) | 0,006 |
| Cardiopathy | 3,05 (1,10 - 8,44) | 0,031 |
| Diabetes mellitus | 2,49 (1,11 - 5,59) | 0,026 |
| Pulmonar disease | 0,51 (0,11 - 2,31) | 0,385 |
| Kidney disease | 2,03 (0,36 - 11,38) | 0,421 |
| Systemic arterial hypertension | 2,36 (1,28 - 4,34) | 0,006 |
| Obesity | 1,76 (0,83 - 3,72) | 0,138 |
| Other | 1,68 (0,92 - 3,07) | 0,094 |
| **Number of comorbidities** |  | 0,005 |
| 1 comorbidity | 2,28 (0,93 - 5,57) | 0,070 |
| 2 or more comorbidities | 4,04 (1,72 - 9,48) | 0,001 |
| **Smoking** | 0,47 (0,11 - 2,13) | 0,329 |
| **Abdominal circumference (cm)** | 1,01 (0,98 - 1,04) | 0,466 |
| **Weight (kg)** | 1,01 (0,98 - 1,04) | 0,444 |
| **BMI (kg/m^2^)** | 1,07 (0,99 - 1,15) | 0,096 |
| **SBP (mmHg)** | 1,02 (1,01 - 1,04) | 0,025 |
| **DBP (mmHg)** | 1,03 (1,01 - 1,06) | 0,088 |
| **Symptoms** |  |  |
| Asymptomatic | 0,27 (0,03 - 2,11) | 0,212 |
| Skin manifestations | 0,56 (0,07 - 4,66) | 0,592 |
| Arthralgia | 1,62 (0,88 - 2,98) | 0,118 |
| Asthenia | 1,09 (0,60 - 1,97) | 0,784 |
| Headache | 0,84 (0,46 - 1,55) | 0,588 |
| **Rhinorrhea** | 0,79 (0,40 - 1,55) | 0,493 |
| Diarrhoea | 1,08 (0,58 - 2,00) | 0,817 |
| Dyspnoea | 4,85 (2,51 - 9,37) | <0,001 |
| Fever | 1,24 (0,68 - 2,26) | 0,485 |
| Myalgia | 2,03 (1,10 - 3,75) | 0,024 |
| Nausea | 1,39 (0,72 - 2,70) | 0,332 |
| Anosmia | 0,35 (0,19 - 0,66) | 0,001 |
| Ageusia | 0,44 (0,24 - 0,81) | 0,009 |
| Dizziness | 0,68 (0,3 - 1,54) | 0,349 |
| Cough | 2,44 (1,28 - 4,64) | 0,006 |
| Vomit | 2,28 (1,13 - 4,63) | 0,022 |
| Other symptoms | 0,87 (0,43 - 1,78) | 0,709 |
| **Disease-modifying antirheumatic or immussupressive drugs** |  |  |
| Without treatment | 0,79 (0,09 - 6,93) | 0,833 |
| Abatacept | 2,47 (0,57 - 10,68) | 0,226 |
| Anti-IL12/23 | 0,00 (-) | 1,000 |
| Anti-TNF | 0,38 (0,17 - 0,84) | 0,017 |
| Azathioprine | (-) | 0,999 |
| Corticosteroids (oral) |  | 0,007 |
| No use | 1,98 (1,02 - 3,81) | 0,042 |
| < 10 mg/day n (%) | 6,69 (2,08 - 21,48) | 0,001 |
| ≥ 11 to 20 mg/day n (%) | 2,87 (0,25 - 32,75) | 0,397 |
| Hydroxychloroquine/Chloroquine | 0,94 (0,34 - 2,63) | 0,910 |
| JAKi | 1,12 (0,40 - 3,16) | 0,835 |
| Leflunomide | 1,10 (0,55 - 2,17) | 0,791 |
| Methotrexate |  | 0,783 |
| ≤ 20 mg/week | 0,81 (0,42 - 1,55) | 0,522 |
| ≥ 21 mg/week | 1,08 (0,37 - 3,14) | 0,894 |
| Rituximab | 1,64 (0,49 - 5,45) | 0,419 |
| Sulfasalazin | 2,03 (0,36 - 11,38) | 0,421 |
| Tocilizumab | 0,81 (0,29 - 2,24) | 0,687 |
| Other | 0,58 (0,22 - 1,58) | 0,288 |
| scDMARD | 1,05 (0,54 - 2,05) | 0,878 |
| bDMARD | 0,58 (0,32 - 1,08) | 0,085 |
| Treatment suspension | 2,27 (1,20 - 4,29) | 0,011 |
| Rheumatoid factor |  | 0,513 |
| Negative | 1,67 (0,70 - 4,01) | 0,248 |
| Positive | 0,00 (-) | 0,998 |
| **Anti-CCP - negative** | 1,82 (0,65 - 5,12) | 0,258 |
| **Erosive disease** | 2,00 (0,89 - 4,48) | 0,092 |
| **Extra articular manifestations** | 2,89 (1,13 - 7,41) | 0,027 |

(-) without precision

OR - odds ratio

95% CI - 95% confidence interval

BMI: Body mass index

SBP: systolic blood pressure

DBP: diastolic blood pressure

bDMARD: biologic DMARD

scDMARD: sinthetic conventional DMARD

JAKi: Janus Kinase inhibitor
